# Dietary habits among primary school learners in the Tshwane West District of Gauteng, South Africa

**DOI:** 10.4102/hsag.v29i0.2746

**Published:** 2024-11-06

**Authors:** Morentho C. Phetla, Linda Skaal, Kiprano P. Chelule

**Affiliations:** 1 Department of Human Nutrition and Dietetics, School of Health Care Sciences, Sefako Makgatho Health Sciences University, Pretoria, South Africa; 2 Department of Public Health, School of Health Care Sciences, Sefako Makgatho Health Science University, Pretoria, South Africa

**Keywords:** learners, primary school, dietary habits, nutrition status, nutrition, obesity

## Abstract

**Background:**

Poor dietary habits are major contributors to malnutrition globally, particularly in children living in African countries. The widespread transition from African diet of healthy indigenous foods to a nutrient-poor Western-style diet is well-documented in global societal culture.

**Aim:**

This study aimed to assess the dietary habits and their nutritional implications among learners in public primary schools.

**Setting:**

City of Tshwane, located in the Gauteng province of South Africa.

**Methods:**

This was a quantitative cross-sectional study where researcher-administered questionnaire was used to collect data from 814 primary school learners in grades 4–7. Anthropometric data were also collected. The study was conducted in 10 primary schools in the City of Tshwane.

**Results:**

Unhealthy dietary practices were observed where consumption of refined carbohydrates, sugar-filled beverages and limited protein was prevalent. The prevalence rates for overweight and obesity were 15.1% and 11.3%, respectively. Most learners (77.4%) purchased foods from street vendors and tuckshops. Also, learners who knew about healthy eating were less likely to be underweight than those who did not (OR: 0.35; 95% CI: 0.14–0.85; *p* = 0.020).

**Conclusion:**

Poor dietary habits are demonstrated in this study and may be associated with the rising levels of overweight and obesity among the learners. The findings also showed that the school environment is the main source of exposure to unhealthy diet.

**Contribution:**

Intervention strategies, such as amendment of the national school nutrition policy, need to be implemented.

## Introduction

Poor dietary habits lead to malnutrition, which is a major public health concern contributing to the global burden of disease. It is reported that a significant number of children suffer from malnutrition because of poor eating habits worldwide (UNICEF 2019). Evidence shows that poor eating habits have been linked to life-threatening consequences such as stroke, type 2 diabetes mellitus (T2DM), different types of cancers and cardiovascular diseases in both children and adults (World Health Organization [WHO] 2023). The documented benefits of good nutrition include a delayed onset of chronic diseases of lifestyle (Centre for Disease prevention 2021).

Globalisation and urbanisation have exposed people to unhealthy, Westernised diets that are high in fats, sugars and refined carbohydrates while lacking micronutrients, fibre and whole foods (Casari et al. 2022). Highly processed foods, which contribute to the global obesity epidemic, are firm favourites among children and adolescents (WHO 2019). The disadvantages of poor nutrition are well-documented, but it is crucial for children to be taught about healthy eating regularly so that they may adopt good dietary practices early in life and carry these over into adulthood. Assessing learners’ daily dietary habits may give researchers an indication of whether they are eating healthily or not, and may contribute to the development of school-based interventions to promote healthy diets and prevent the short- and long-term consequences of poor eating habits from developing among learners.

Studies conducted in developed and developing countries have reported that the consumption of fruits and vegetables among primary school learners is low (Hong & Piaseu 2017). A recent study conducted in urban South African schools found that learners purchased unhealthy foods from street vendors and school tuckshops (Phetla & Skaal 2023). Phetla and Skaal (2023) in their study conducted in Tshwane, South Africa, further reported that the lunchboxes of most learners contained sweets, sugar-sweetened beverages and snacks, while only a few contained fruits and vegetables. Carrying a nutritious lunchbox to school contributes to good eating habits and the development of a healthy body and mind (Nathan et al. 2019). It is therefore concerning that most learners carried lunchboxes containing unhealthy food to school (Sutherland et al. 2020). Learners who brought pocket money to purchase food from school tuckshops and street vendors were prone to buying fast foods containing high fat, high salt, refined carbohydrates, high sugar and low protein (Okeyo et al. 2020). Studies show that primary school learners are exposed to the influence of fast food and take-away establishments because of their aggressive marketing and easy accessibility (Scully et al. 2020). The statistics on childhood health in South Africa indicate a progressive increase in the number of obese children (Nwosu et al. 2022). Could this be because of poor dietary habits? Do dietary habits affect the nutritional status of learners? The aim of this study was to assess the dietary habits of learners in public primary schools in the Tshwane West District of the Gauteng province in South Africa. The hypothesis of the study was that poor dietary habits were being practised by learners attending public primary schools in the Tshwane West District of Gauteng, South Africa.

## Research methods and design

### Study design and setting

The authors used a quantitative approach and a cross-sectional descriptive study design to address the question: *What are the dietary habits of primary school learners in 10 public primary schools in the Tshwane West District of Gauteng, South Africa?* Data were collected at schools during breaks or life orientation periods for 4 months (March 2021–June 2021). School principals provided conducive areas for data collection. The multistage sampling technique was applied to sample 814 learners from 10 primary schools in the Tshwane West District. Primary schools were stratified according to socioeconomic status (SES), namely low SES, middle SES and high SES (affluent), to resemble the entire population. Two schools were randomly selected from each SES to participate in the study. Morgan and Krejcie’s sample size table was used to determine a suitable sample size, using the following formula (see Equation 1):

$$n=\frac{X^{2}NP(1-P)}{d^{2}(N-1)+X^{2}P(1-P)}$$ [Eqn 1]

Where:

*N* = population size

*X*^2^ = table value of Chi-square at 95% CI

*P* = population proportion (0.50)

*d* = degree of accuracy.

Here, the minimum sample size was 359. The authors included 814 learners so that internal and external validity could be better determined. It was possible to increase the sample size because most of the learners managed to bring back the parental consent forms.

### Study population and sampling

A total of 814 learners (boys = 360; girls = 454) from grades 4 to 7 participated in the study. The authors used the stratified random sampling technique to select the schools. Simple random sampling was used to select participants from 10 primary schools. The eligibility criteria included participants who were in grades 4 to 7 and whose parents had signed an informed consent and learners signed assent forms. Exclusion criteria were learners whose parents or guardians did not sign parental consent forms.

### Data collection

Data were collected by the researcher and research assistants between March and June 2021. The school principals provided suitable spaces for data collection. The questionnaire was written in English and translated to Setswana, which is the home language of most primary school learners in the Tshwane West District of Gauteng. The questionnaire included sociodemographic data as well as questions about the food consumed by learners at home and at school. The development of questions was guided by the South African Food-Based Dietary Guidelines (SAFBDGs). After completing the questionnaire, the learners’ anthropometric (weight and height) measurements were taken.

### Data analysis

Data were analysed using the statistical software package Stata version 17. AnthroPlus software was used to calculate and classify body mass index (BMI) for age, height for age (low height for age indicates stunting) and weight for age (low weight for age indicates underweight). Descriptive statistics were analysed using frequency distribution, standard deviation (s.d.) and mean. Logistic regression was applied to analyse the sociodemographic factors associated with BMI for age, height for age (stunting) and weight for age (wasting). The *p*-values less than 0.05 indicated the association.

### Ethical considerations

The study was conducted in accordance with the Declaration of Helsinki and approved by the Turfloop Review Board (TREC/343/2019:PG). The permission to conduct this study was sought and granted from the National Department of Basic Education (DBE). The learners were provided with a participants’ information leaflet to give their parents and guardians. It was explaining the purpose, aims and objectives of the study. The parents or guardians of the participants gave their written consent, and the participating learners gave their assent. Learners were informed that they had the right to withdraw from the study without being penalised. Privacy and confidentiality were maintained.

## Results

### Sociodemographic profile of participants

Table 1 indicates the sociodemographic characteristics of the learners, the majority of whom were girls (55.8%). There was an almost equal number of learners under 11 years (49%) and older than 11 years (51%), with mean = 11.3 and s.d. = 1.4 years. Nearly a third of the learners were in grade 7 (30.2%). More than half of the learners resided in formal urban areas (56.8%), while 42.4% resided in peri-urban areas. Close to half (45.8%) of the learners carried lunch boxes to school. More than half (52.3%) of the learners received meals from the feeding programmes at their schools.

**TABLE 1:** Sociodemographic characteristics of the participants (*N* = 814).

Sociodemographic characteristics	*n*	%
**Gender**		
Boy	360	55.8
Girl	454	44.2
**Age (years)**		
< 11	399	49.0
> 11	415	51.0
**Urban type of residence**		
Peri-urban	352	43.2
Formal urban	462	56.8
**A learner stays with**		
Parents	605	74.3
Other family members	209	25.7
**Learner carries lunchbox to school**		
Yes	441	45.8
No	373	54.2
**Learner eats from the school feeding programme**		
Yes	426	52.3
No	388	47.7

Table 2 indicates that the majority (70%) of the learners had normal weight, 15.1% were overweight and 11.3% were obese. It also shows that 46.4% of the learners had normal height while 45.6% were at risk of stunting. However, 3.4% of learners were in the stunting category.

**TABLE 2:** Body mass index and height based on age- and gender-specific cutoff points.

BMI	%	Height	%
Obese	11.3	Severely stunted	0.5
Overweight	15.1	Moderately stunted	2.9
Normal weight	70.0	Risk of stunting	45.6
Moderate underweight	3.1	Normal height	46.4
Severe underweight	0.5	Tall	3.8
-	-	Very tall	0.7

BMI, body mass index.

Table 3 shows that the majority (78.9%) of the learners ate breakfast and half (49.9%) of these ate breakfast every day. Nearly half (48.8%) of the learners ate cereal in the morning. For those learners who skipped breakfast, a lack of time was the main reason (36.8%). The majority (77.4%) of the learners bought food or snacks at school, and almost half (45.6%) bought food from street vendors. The majority (68.1%) of the learners bought food or snacks at school during break and only a few (23.6%) bought them every school day. Less than half (39.4%) of the learners carried pocket money every school day.

**TABLE 3:** The frequency distribution of eating breakfast and carrying pocket money.

Variables	*n*	%
**Eat breakfast before going to school (*n* = 814)**		
No	172	21.1
Yes	642	78.9
**Breakfast food (*n* = 642)**		
Cereal	313	48.8
Bread	181	28.2
Soft porridge	127	19.7
Others	21	3.3
**Frequency of eating breakfast (*n* = 642)**		
School days	65	10.1
Everyday	320	49.9
Sometimes	257	40.0
**Reason for not eating breakfast daily (*n* = 494)**		
Lack time	182	36.8
No reason stated	95	19.2
Not hungry	109	22.0
No food	108	21.9
**The child buys food at school (*n* = 814)**		
No	184	22.6
Yes	630	77.4
**Outlet where the learner buys at school (*n* = 630)**		
School tuckshop	180	28.6
Street vendor	287	45.6
Street vendor and tuckshop	163	25.9
**Carry pocket money (*n* = 814)**		
No	184	22.6
Yes	630	77.4

Table 4 shows that almost all learners (98.9%) were consuming fruits, with the majority (86.2%) consuming less than four fruits daily. Most learners never ate fruits (64.5%) or vegetables (47.2%) because they were not available. Most of the learners (95%) consumed vegetables, with most (87.4%) consuming less than four vegetables per day, and over a third (34.9%) of the learners consumed vegetables every day.

**TABLE 4:** The frequency distribution of fruits and vegetables consumption.

Variables	*n*	%
**Eat fruits (*n* = 814)**		
No	9	1.1
Yes	805	98.9
**Number of fruits eaten daily (*n* = 805)**		
Less than four	694	86.2
More than four	111	13.8
**Frequency of eating fruits (*n* = 805)**		
Daily	278	34.5
Weekends	59	7.3
Sometimes	468	58.1
**Reasons for not eating fruits daily (*n* = 536)**		
Not available	348	64.9
Expensive	50	9.3
Dislike fruits	60	11.2
Not necessary	11	2.0
No reason	67	12.5
**Eat vegetables (*n* = 814)**		
No	41	5.0
Yes	773	95.0
**Number of vegetables eaten daily (*n* = 773)**		
Less than four	676	87.4
More than four	97	12.5
**Frequency of eating vegetables (*n* = 773)**		
Daily	270	34.9
Weekends	162	20.9
Sometimes	341	44.1
**Reasons for not eating vegetables daily (*n* = 544)**		
Not available	257	47.2
Expensive	45	8.3
Dislike	110	20.2
Not necessary	19	3.5
No reason	113	20.8

### Sociodemographic and food habits associated with stunting

Bivariate logistic regression was used to determine the relationship between sociodemographic data, healthy eating knowledge and stunting status. Learners over 11 years were three times more likely to be stunted than those who were younger (OR: 2.8; 95% CI: 1.18–6.78; *p* = 0.019). Participating learners who consumed breakfast everyday had a lower prevalence of stunting than those who did not have breakfast before going to school (OR: 2.4; 95% CI: 1.06–5.59; *p* = 0.035). The rest of the factors did not show any association with stunting (Table 5). However, the rate of stunting in this cohort of children was low at 3.4%.

**TABLE 5:** Factors associated with stunting.

Variable	Details	%	OR	95% CI	*p*
Gender	Girl	44.2	1.00	1.00	1.000
	Boy	55.8	0.62	0.29–1.35	0.230
Age category (years)	< 11	49.0	1.00	1.00	1.000
	> 11	51.0	2.80	1.18–6.78	0.019*
Eat breakfast	No	52.7	1.00	1.00	1.000
	School days	7.7	1.50	0.32–7.24	0.590
	Everyday	39.6	2.40	1.06–5.59	0.035*
School feeding programme	No	47.7	1.00	1.00	1.000
	Yes	52.3	0.72	0.33–1.55	0.400
Taught healthy eating	No	10.9	1.00	1.00	1.000
	Yes	89.1	1.55	0.36–6.67	0.550
Perform sports at school	No	50.9	1.00	1.00	1.000
	Yes	49.1	0.81	0.38–1.73	0.590
Eat vegetables	No	6.4	-	-	-
	Yes	93.6	0.50	0.14–1.70	0.270
Eat fruits	No	5.6	1.00	1.00	1.000
	Yes	94.4	1.36	0.24–7.67	0.730
Play outside	No	58.2	1.00	1.00	1.000
	Yes	41.8	1.30	0.6–2.81	0.500
Carry lunchbox	No	45.8	1.00	1.00	1.000
	Yes	54.2	1.06	0.49–2.29	0.880

Note: *, Signifies significance @95% CI.

Sociodemographic and food habits associated with underweight and overweight Bivariate logistic regression was used to determine the relationship between sociodemographic data, healthy eating knowledge and underweight status. Learners who were taught about healthy eating habits were less likely to be underweight compared to those who were not taught (OR: 0.35; 95% CI: 0.14–0.85; *p* = 0.020). None of the other factors showed any association with underweight (Table 6). Similarly, no factors identified in this study showed any association with overweight.

**TABLE 6:** Factors associated with underweight.

Variable	Details	%	OR	95% CI	*p*
Gender	Girl	44.2	1.00	1.00	1.000
	Boy	55.8	1.23	0.57–2.67	0.590
Age category (years)	< 11	49.0	1.00	1.00	1.000
	> 11	51.0	2.10	0.92–4.65	0.075
Eat breakfast often	No	52.7	1.00	1.00	1.000
	School days	7.7	0.37	0.048–2.8	0.330
	Everyday	39.6	0.27	0.29–1.40	0.310
School feeding programme	No	47.7	1.00	1.00	1.000
	Yes	52.3	1.05	0.49–2.24	0.890
Taught healthy eating	No	10.9	1.00	1.00	1.000
	Yes	89.1	0.35	0.14–0.85	0.020*
Perform sports at school	No	50.9	1.00	1.00	1.000
	Yes	49.1	0.56	0.26–1.24	0.150
Eat vegetables	No	6.4	1.00	1.00	1.000
	Yes	93.6	0.66	0.20–3.82	0.870
Eat fruits	No	5.6	1.00	1.00	1.000
	Yes	94.4	1.64	0.22–12.34	0.630
Play outside	No	58.2	1.00	1.00	1.000
	Yes	41.8	1.90	0.89–4.1	0.098
Carry lunch box	No	45.8	1.00	1.00	1.000
	Yes	54.2	0.84	0.39–1.79	0.650

Note: *, Signifies significance @95% CI.

## Discussion

The aim of this study was to determine the dietary habits and nutritional status of school learners. The rate of stunting was low (3.4%) and within the WHO recommended reference value ranges (WHO 2022). However, close to a quarter of the learners were overweight and obese, with the figures being above those reported in previous literature (Casari et al. 2022). This finding is not unique to Tshwane West or Gauteng. Studies conducted in other provinces in South Africa have reported similar findings (Negash et al. 2017; Otitoola et al. 2021). A systematic review and meta-analysis conducted in African countries, including Ghana, Tunisia, Uganda and Morocco, indicated an overall estimate of 9.5% – 11% of overweight and 4% – 6.9% obesity among learners (Ochola & Masibo 2014). These findings confirm that poor dietary habits negatively affect the nutritional status of learners.

Urbanisation is characterised by limited access to healthy foods, which is demonstrated by learners having easy access to nutrient-poor food such as bunny chows and deep-fried chips from tuckshops and street vendors (Garcia et al. 2018). It is not surprising that the current study found that primary school learners had poor eating habits. Other studies reported similar findings in South Africa, where poor eating habits were found in urban settings (Nkambule et al. 2019; Ochola & Masibo 2014). Global studies have yielded interesting results. Russia reported a high consumption of ultra-processed foods and confectionery among learners (Podchinenova et al. 2023). Learners in a study conducted in Saudi Arabia reported that they skipped meals, and only half of them ate breakfast daily. This is a public health concern because breakfast is considered an important meal of the day, providing energy to children for optimal performance of activities of daily living (Wijayanti et al. 2017). Skipping breakfast is associated with the development of obesity (Ma et al. 2020).

The current study revealed that approximately half of the learners carried pocket money to school and used it to buy unhealthy food from street vendors and tuckshop outlets. Previous studies conducted in South Africa indicated that more than 50% of schoolchildren carried pocket money and used it to buy unhealthy food from street vendors (Faber & Fonseca 2014). Several studies showed a significant association between pocket money and obesity, as learners tend to buy what is readily available and affordable, without considering the nutritional value of the items purchased (Güven & Öncü 2022).

The findings of the current study indicate that most of the foods purchased at the school tuckshop and street vendors were unhealthy food items, such as bunny chows, fried chips, fat cakes, cookies and sweets. Documented reasons for the purchase of unhealthy foods include the fact that children are influenced by the sweet taste, availability and affordability of these foods and snacks (Kigaru et al. 2015). The current study found a significant association between underweight and learning about healthy foods. Further studies are needed to investigate why food outlets around schools continue to sell unhealthy foods. Street vendors and tuckshop operators need to be educated about the sale of tasty, healthy snacks with a long shelf life. The researcher recommends that schools communicate the National School Nutrition Programme Guidelines for Tuck Shop Operators and monitor the goods sold by street vendors and tuckshop operators as stipulated in the DBE policy.

Although most learners in the current study reportedly consumed fruits and vegetables every day, the portions were less than four per day. This consumption rate did not meet the recommendation of the SAFBDGs (Temple & Steyn 2013). The most cited reason they provided for not eating enough fruits and vegetables was their lack of availability. The lack of fruits and vegetables in the school feeding programme also deviates from the DBE guidelines, which stipulate that the school feeding programme should always provide at least one fruit to learners per day. For a long time, parents have struggled to force children to consume vegetables; in the current study, approximately 20% of learners reported that they dislike vegetables and therefore do not consume them daily. The researcher recommends that schools run campaigns like ‘bring your own vegetable to school’ to create a hype around healthy eating among learners.

In New Zealand and Spain, barriers to eating vegetables and fruits were recorded as follows: very expensive, no time to prepare vegetables and poor cooking skills compared to buying junk foods from food outlets (Karki et al. 2019). Studies have also shown that fast food marketing is aggressive, compared to a scant marketing of the benefits of consuming fruits and vegetables (Draper et al. 2019). Interestingly, most learners consumed fruits and vegetables, albeit not as frequently as they should. A low consumption of fruits and vegetables was recorded in Thailand and Kenya (Kigaru et al. 2015) despite evidence that the consumption of fruits and vegetables prevents cancer, cardiovascular disease, T2DM, obesity and adiposity among school-age children (Naudé 2013).

### Study limitations

The study relied entirely on the self-reported food habits of learners. The reliability of the data may therefore be interpreted with caution, particularly when seeking the relationship between these food habits and nutritional status.

## Conclusion and recommendations

Poor dietary habits are evident in this cohort of learners, which ultimately affect their nutritional status. The school environment plays an enormous role in exposing learners to poor dietary habits. Therefore, it is recommended that school nutrition programmes are implemented by dietitians. The DBE should collaborate with the Nutrition Directorate, universities, and Department of Agriculture, to craft a user-friendly nutritional programme that encourages healthy dietary practices.

## References

[CIT0001] Casari, S., Di Paola, M., Banci, E., Diallo, S., Scarallo, L., Renzo, S. et al., 2022, ‘Changing dietary habits: The impact of urbanization and rising socio-economic status in families from Burkina Faso in Sub-Saharan Africa’, *Nutrients* 14(9), 1782. 10.3390/nu14091782 35565752 PMC9104313

[CIT0002] Centre for Disease prevention, 2021, *Division of nutrition, physical activity and obesity*, viewed 18 August 2023, from https://www.cdc.gov/nccdphp/dnpao/index.html.

[CIT0003] Draper, C., Tomaz, S., Bassett, S., Harbron, J., Kruger, H., Micklesfield, L. et al., 2019, ‘Results from the healthy active kids South Africa 2018 report card’, *South African Journal of Child Health* 13(3), 130–136. 10.7196/SAJCH.2019.v13i3.1640

[CIT0004] Faber, J. & Fonseca, L.M., 2014, ‘How sample size influences research outcomes’, *Dental Press Journal of Orthodontics* 19(4), 27–29. 10.1590/2176-9451.19.4.027-029.ebo PMC4296634 25279518

[CIT0005] Garcia, M.T., Sato, P.M., Trude, A.C.B., Eckmann, T., Steeves, E.T.A., Hurley, K.M. et al., 2018, ‘Factors associated with home meal preparation and fast-food sources use among low-income urban African American adults’, *Ecology of Food and Nutrition* 57(1), 13–31. 10.1080/03670244.2017.1406853 29227695 PMC5884711

[CIT0006] Güven, Y. & Öncü, E., 2022, ‘The relationship between junk food consumption, healthy nutrition, and obesity among children aged 7 to 8 years in Mersin, Turkey’, *Nutrition Research* 103, 1–10. 10.1016/j.nutres.2022.03.004 35429797

[CIT0007] Hong, S.A. & Piaseu, N., 2017, ‘Prevalence and determinants of sufficient fruit and vegetable consumption among primary school children in Nakhon Pathom, Thailand’, *Nutrition Research and Practice* 11(2), 130–138. 10.4162/nrp.2017.11.2.130 28386386 PMC5376531

[CIT0008] Karki, A., Shrestha, A. & Subedi, N., 2019, ‘Prevalence and associated factors of childhood overweight/obesity among primary school children in urban Nepal’, *BMC Public Health* 19, 1–12. 10.1186/s12889-019-7406-9 31387571 PMC6685156

[CIT0009] Kigaru, D.M.D., Loechl, C., Moleah, T., Macharia-Mutie, C. & Ndungu, Z.W., 2015, ‘Nutrition knowledge, attitude and practices among urban primary school children in Nairobi City, Kenya: A KAP study’, *BMC Nutrition* 1, 1–8. 10.1186/s40795-015-0040-8

[CIT0010] Ma, X., Chen, Q., Pu, Y., Guo, M., Jiang, Z., Huang, W. et al., 2020, ‘Skipping breakfast is associated with overweight and obesity: A systematic review and meta-analysis’, *Obesity Research & Clinical Practice* 14(1), 1–8. 10.1016/j.orcp.2019.12.002 31918985

[CIT0011] Nathan, N., Janssen, L., Sutherland, R., Hodder, R.K., Evans, C.E., Booth, D. et al., 2019, ‘The effectiveness of lunchbox interventions on improving the foods and beverages packed and consumed by children at centre-based care or school: A systematic review and meta-analysis’, *International Journal of Behavioral Nutrition and Physical Activity* 16, 1–15. 10.1186/s12966-019-0798-1 31036038 PMC6489330

[CIT0012] Naudé, C.E., 2013, ‘“Eat plenty of vegetables and fruit every day”: A food-based dietary guideline for South Africa’, *South African Journal of Clinical Nutrition* 26, S46–S56.

[CIT0013] Negash, S., Agyemang, C., Matsha, T.E., Peer, N., Erasmus, R.T. & Kengne, A.P., 2017, ‘Differential prevalence and associations of overweight and obesity by gender and population group among school learners in South Africa: A cross-sectional study’, *BMC Obesity* 4, 29. 10.1186/s40608-017-0165-1 28725448 PMC5514529

[CIT0014] Nkambule, N.R., Madiba, T.K. & Bhayat, A., 2019, ‘Dental caries, body mass index, and diet among learners at selected primary schools in Pretoria, Gauteng Province, South Africa’, *Journal of Contemporary Dental Practice* 20(11), 1241–1248. 10.5005/jp-journals-10024-2685 31892673

[CIT0015] Nwosu, E., Fismen, A.-S., Helleve, A., Hongoro, C., Sewpaul, R., Reddy, P. et al., 2022, ‘Trends in prevalence of overweight and obesity among South African and European adolescents: A comparative outlook’, *BMC Public Health* 22, 2287. 10.1186/s12889-022-14724-2 36474229 PMC9727950

[CIT0016] Ochola, S. & Masibo, P.K., 2014, ‘Dietary intake of schoolchildren and adolescents in developing countries’, *Annals of Nutrition and Metabolism* 64, 24–40. 10.1159/000365125 25341871

[CIT0017] Okeyo, A.P., Seekoe, E., De Villiers, A., Faber, M., Nel, J.H. & Steyn, N.P., 2020, ‘The food and nutrition environment at secondary schools in the Eastern Cape, South Africa as reported by learners’, *International Journal of Environmental Research and Public Health* 17(11), 4038. 10.3390/ijerph17114038 32517072 PMC7312062

[CIT0018] Otitoola, O., Oldewage-Theron, W. & Egal, A., 2021, ‘Prevalence of overweight and obesity among selected schoolchildren and adolescents in Cofimvaba, South Africa’, *South African Journal of Clinical Nutrition* 34(3), 97–102. 10.1080/16070658.2020.1733305

[CIT0019] Phetla, M.C. & Skaal, L., 2023 ‘Scanning for obesogenicity of primary school environments in Tshwane, Gauteng, South Africa’, *International Journal of Environmental Research and Public Health* 20, 6889. 10.3390/ijerph20196889 37835158 PMC10572655

[CIT0020] Podchinenova, D.V., Tabakaeva, O.V., Ogorodova, L.M., Samoilova, I.G., Matveeva, M.V. & Oleynik, O.A., 2023, ‘Patterns of eating habits and body composition in primary school children’, *Vopr Pitan* 92, 45–53. 10.33029/0042-8833-2023-92-3-45-53 37432706

[CIT0021] Scully, M., Morley, B., Niven, P., Crawford, D., Pratt, I.S., Wakefield, M. et al., 2020, ‘Factors associated with frequent consumption of fast food among Australian secondary school students’, *Public Health Nutrition* 23(8), 1340–1349. 10.1017/S1368980019004208 32172726 PMC10200663

[CIT0022] Sutherland, R., Nathan, N., Brown, A., Yoong, S., Reynolds, R., Walton, A. et al., 2020, ‘A cross-sectional study to determine the energy density and nutritional quality of primary-school children’s lunchboxes’, *Public Health Nutrition* 23, 1108–1116. 10.1017/S1368980019003379 31969199 PMC10200590

[CIT0023] Temple, N.J. & Steyn, N.P., 2013, ‘Sugar and health: A food-based dietary guideline for South Africa’, *South African Journal of Clinical Nutrition* 26, S100–S104.

[CIT0024] UNICEF, 2019, *The state of the World Children 2019*, UNICEF, viewed 10 July 2022, from https://www.unicef.org/reports/state-of-worlds-children-2019.

[CIT0025] Wijayanti, A.E., Lusmilasari, L. & Claramita, M., 2017, ‘Promoting healthy food education for elementary school children at post Merapi eruption area of Cangkringan district, Yogyakarta, Indonesia: A quasi experimental study using “learning with fun” approach’, *Journal of Nursing Education and Practice* 7(3), 128–136. 10.5430/jnep.v7n3p128

[CIT0026] World Health Organization (WHO), 2019, *Global action plan on physical activity 2018–2030: More active people for a healthier world*, World Health Organization, Geneva.

[CIT0027] World Health Organization (WHO), 2022, *Prevalence of stunting in children under 5 (%)*, viewed 18 August 2023, from https://www.who.int/data/gho/data/indicators/indicator-details/GHO/gho-jme-stunting-prevalence.

[CIT0028] World Health Organization (WHO), 2023, *Healthy diet*, Geneva, viewed 2023, from https://www.who.int/initiatives/behealthy/healthy-diet#:~:text=A%20healthy%20diet,for%20healthy%20diet.

