# Corrigendum: Dietary habits among primary school learners in the Tshwane West District of Gauteng, South Africa

**DOI:** 10.4102/hsag.v30i0.2989

**Published:** 2025-02-25

**Authors:** Morentho C. Phetla, Linda Skaal, Paul K. Chelule

**Affiliations:** 1 Department of Human Nutrition and Dietetics, School of Health Care Sciences, Sefako Makgatho Health Science University, Pretoria, South Africa; 2 Department of Public Health, School of Health Care Sciences, Sefako Makgatho Health Science University, Pretoria, South Africa

In the original article published, Phetla, M.C., Skaal, L. & Chelule, K.P., 2024, ‘Dietary habits among primary school learners in the Tshwane West District of Gauteng, South Africa’, *Health SA Gesondheid* 29(0), a2746. https://doi.org/10.4102/hsag.v29i0.2746, an author name was incorrectly spelt.

Instead of:

Kiprano P. Chelule

It should be:

Paul Kiprono Chelule

The authors apologise for this error.

The correction does not change the study’s findings, its significance or overall interpretation of its results or the scientific conclusions of the article in any way.

